# Selective Product Enhancement in an Auger Reactor: Pyrolysis of Pine Bark through In Situ Recirculation of Pyrolysis Vapors

**DOI:** 10.1021/acs.energyfuels.5c06037

**Published:** 2026-02-19

**Authors:** Yusuf Tolunay Kilic, Marcelo Dal Belo Takehara, Øyvind Skreiberg, Kentaro Umeki

**Affiliations:** † Division of Energy Science, 5185 Luleå University of Technology, Luleå SE-97187, Sweden; ‡ Department of Thermal Energy, SINTEF Energy Research, Trondheim NO-7465, Norway

## Abstract

In biomass pyrolysis, final product selectivity is governed not only by major reaction conditions like temperature and heating rate but also by complex vapor–solid interactions and secondary reactions. Yet, the influence of internal flow configuration on pyrolysis vapor remains poorly understood in continuous pyrolysis systems. This study aims to evaluate how controlled vapor–solid interactions via changes in the vapor outlet port location affect the distribution and transformation of pyrolysis products. Experiments were performed in a continuous laboratory-scale auger reactor, processing pine bark at highest treatment temperatures (HTT) of 600, 700, and 800 °C. The reactor featured five independently heated zones and six selectable vapor outlet ports, enabling three vapor flow modes: parallel flow (PF, conventional cocurrent flow operation) and two counterflow (CF) configurations to systematically manipulate vapor–solid contact. Results showed that one of the CF configurations, where vapors passed through the coldest (the incoming) biomass zone before exiting, enhanced vapor condensation on incoming biomass and promoted secondary reactions, leading to up to a 15.5% relative increase in biochar yield compared to PF. The increase in biochar yield was accompanied by an increase in fixed carbon yield, and H_2_ and CH_4_ yields, indicating intensified thermal cracking and polymerization of pyrolysis vapors. In contrast, the CF configuration involving vapor recirculation without interaction with the coldest zone favored external condensation and achieved the highest bio-oil recovery. The PF configuration exhibited the lowest char yield and the highest unaccounted carbon fraction due to poor vapor condensation at elevated outlet temperatures. These findings demonstrate that the manipulation of vapor–solid interactions serves as a critical parameter for steering pyrolysis pathways toward targeted product enhancement, offering a scalable approach for optimizing biochar, gas, and bio-oil yields through in situ vapor recirculation.

## Graphical abstract

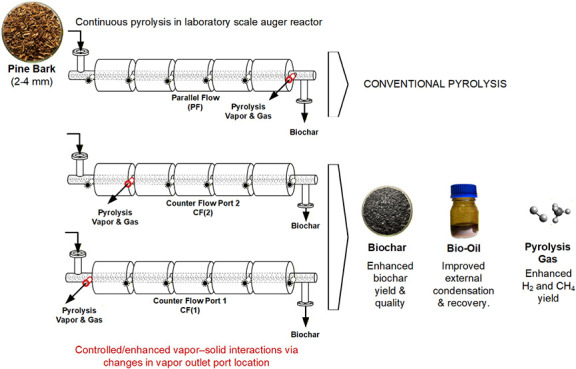

## 1 Introduction

The pyrolysis of lignocellulosic biomass offers a promising route for the thermochemical conversion of renewable feedstocks into energy carriers, value-added chemicals, and functional materials. Among the main products, biochar (also called biocarbon) is often the primary target product due to its growing demand in metallurgical processes,^1^ soil amendment,^2^ and advanced applications such as electrochemical devices.^3^ Liquid-phase products, often called bio-oil, also have application potential to produce fuels, chemicals, and binders.^4^ While biochar yields tend to be low at high temperatures, it is necessary to meet the demands for biochar properties for specific applications, such as high fixed carbon content, achievable at high temperatures.^5,6^ On the other hand, bio-oil is a highly oxygenated and heterogeneous mixture, including heavy molecules that present significant challenges for direct utilization.^7,8^ In this study, we propose the in situ recirculation of pyrolysis vapor inside auger reactors as a potential solution to this technical dilemma.

Previous studies have demonstrated that the liquid-phase products generated during biomass pyrolysis, particularly oxygenated tars, are highly reactive and prone to secondary transformations.^5,9^ Primary pyrolysis intermediates can undergo homogeneous thermal cracking or heterogeneous polymerization on solid surfaces such as biochar or partially devolatilized biomass.^10^ Recent studies have highlighted that the extent and outcome of these transformations can be deliberately varied by controlling the contact between reactive vapor-phase pyrolysis intermediates and the solid. Increasing the residence time, temperature, or surface interactions may promote the conversion of primary pyrolysis vapors into stable volatiles or secondary char, thereby reducing high-molecular-weight fractions in the condensed bio-oil. Several experimental efforts have shown that increasing vapor–solid contact promotes carbonization in biochar, raises fixed carbon content and yield, and suppresses the yield of heavy liquid fractions.^11–16^ These findings support the concept of vapor-phase bio-oil recirculation or addition and thermal transformation of its reactive intermediates within the reactor volume. The concept can serve as an effective strategy to improve product selectivity and mitigate the accumulation of problematic components, such as pyrolytic lignin oligomers in bio-oil.

A variety of strategies have been proposed to exploit these phenomena, including the external recycling of bio-oil fractions onto biomass feedstocks or reactor internals. Huang et al.^14^ demonstrated that sorbing the heavy fraction of bio-oil onto fresh biomass prior to pyrolysis significantly reduced the yield of heavy tar and increased the proportion of light, volatile compounds in the final bio-oil. In a follow-up study, the same group achieved simultaneous enhancement of char and light oil yields through internal recirculation of pyrolytic lignin-rich heavy oil.^13^ Phounglamcheik et al.^12^ reported that embedding bio-oil into woody biomass resulted in synergistic carbon deposition and an 18–19% relative increase in char yield, with negligible impact on char heating value. A study from Veksha et al.^15^ showed that volatile compounds can be deposited onto biochar surfaces and carbonized in situ by guiding pyrolysis vapors through cooler biomass zones in a fixed-bed reactor. As a result, the overall char yield increased without compromising microporosity or adsorption performance. More recently, another study showed that in situ condensation and recycling of volatiles shift carbon retention from vapors to the solid-phase char via repolymerization, increasing char yield significantly.^16^ These findings underscore the role of vapor–solid contact in shaping pyrolysis product yields and motivate the design of reactors that can actively manipulate vapor trajectories.

Despite these promising developments, a critical research gap remains in understanding how secondary reactions, including vapor-phase condensation, cracking, and char deposition, occur dynamically within industrially relevant continuous reactors. Most existing studies have been conducted using thermogravimetric analyzers (TGA), single-particle systems, or batch-type bench-scale fixed-bed reactors. These setups do not necessarily represent the internal gas flow, temperature gradients, and vapor–solid interactions inside industrial scale reactors. In contrast, pilot- and demonstration-scale systems, while more representative of industrial operation, rarely provide systematic analyses of how reactor configuration influences the extent and nature of secondary reactions, particularly in terms of gas–solid contact, temperature distribution, and vapor flow trajectories. These factors represent a critical knowledge gap for successful scale-ups, as they significantly influence the fate of volatiles and the yield and quality of both biochar and bio-oil.

Heat distribution and vapor flows inside industrial reactors are affected by reactor geometry, carrier gas flow characteristics, heat supply methods, and the vapor outlet position, among others.^17,18^ The heating profile is influenced by whether the heat carrier is supplied cocurrent or countercurrent relative to the biomass movement. Meanwhile, the path of evolved volatiles, whether parallel flow or counterflow, can dramatically alter the types and extent of vapor–solid interactions. These differences are not trivial, as they influence the formation and transformation of pyrolysis products, particularly through secondary reactions and condensation of heavy organics. Understanding these differences is essential not only for developing practical and scalable strategies for vapor-phase bio-oil recirculation and upgrading but also for advancing a fundamental understanding of thermochemical conversion. By systematically investigating the effects of reactor design on secondary reactions in situ, the field can advance toward predictive control of pyrolysis product distribution and quality. This knowledge will ultimately inform the design of advanced reactors that capitalize on vapor-phase transformation processes to produce higher-value bio-oil and biochar, contributing to more efficient and tailored biomass conversion technologies.

The present study aims to systematically investigate the role of vapor–solid interactions and in situ recirculation of vapor-phase bio-oil on product distribution and quality during the pyrolysis of pine bark. The investigation was conducted in a custom-designed laboratory-scale continuous auger reactor. By utilizing an auger reactor equipped with five independently controlled heating zones and six selectable outlet ports along the axial positions, we introduced the capability to manipulate the vapor flow trajectories and the degrees of pyrolysis vapor recirculation within the reactor itself. This design allows the examination of how vapor flow direction, ranging from conventional parallel flow to counterflow configurations, affects the extent of pyrolysis vapor condensation and secondary reactions. Unlike prior studies, our approach captures the dynamic and spatially resolved nature of the pyrolysis vapor passage in a continuous auger reactor environment. The results from this study provide a framework for integrating bio-oil recirculation strategies into scalable pyrolysis technologies.

## 2 Material and Methods

### 2.1 Reactor Design and Instrumentation

A continuous laboratory-scale auger pyrolysis reactor (Figure 1) was constructed from a stainless steel tube (grade: 253 MA, 1.33 m heated length, 40.5 mm inner diameter) with an internal screw conveyor (auger). The auger was driven by a variable-speed motor (∼3 rpm in all tests). Five independent heating zones provided an adjustable axial temperature profile from the feed inlet to the biochar outlet. In each heating zone, a pair of semicylindrical ceramic fiber heaters, type VS from Watlow, was controlled by a single channel of the PID controller (HTC-5500 from Hemi Heating AB) using K-type thermocouples located at the outer wall of the reactor. Additionally, six K-type thermocouples (TC1–TC6) were placed with equal spacing (266 mm) inside the reactor at the radial position exactly on the inner wall. TC1 was located just before the first heater (85 mm downstream of the feed entrance), and TC6 was located right after the last heater (85 mm upstream of the biochar discharge). Gas-phase pressure was monitored at three positions (above ports 2, 4, and 6) using pressure transducers PMC 11 from Endress+Hauser (P1–P3, 0–1 bar range, ±0.5% full-scale accuracy).

**Figure 1:**
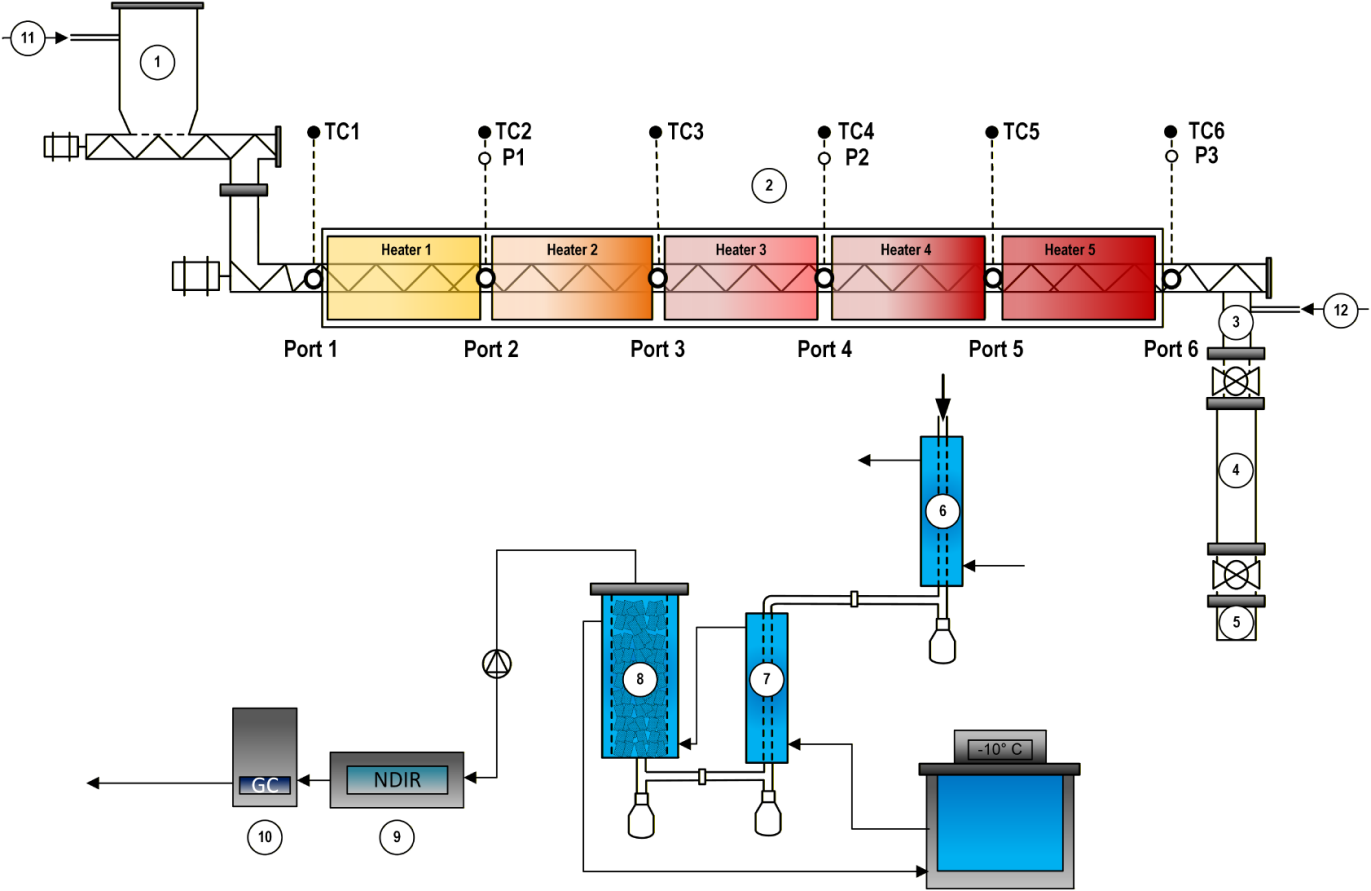
Schematic representation of the lab-scale auger pyrolysis system used in this study. The setup includes a biomass feeder (1), an electrically heated auger reactor (2), a three-stage char collection system (3, 4, 5), a three-stage condenser system (6, 7, 8), and a gas analysis system (9, 10). Additional components include a nitrogen supply (11–12).

### 2.2 Feedstock

The feedstock was pine bark, which was obtained from the Swedish University of Agricultural Sciences (SLU). Bark was milled and sieved to a 2.0–4.0 mm particle size, chosen based on screw-feeder constraints to maintain continuous, stable feeding. A representative 500 g batch was prepared for each run from the same larger batch of feedstock by a rotary sample divider PT 100 from Retsch GmbH (according to the ISO 14780:2017 sample preparation method). The initial moisture content was 8.6 ± 0.8 wt % (measured according to ISO 18134-3). Proximate and ultimate analyses of the bark are given in Table 1.

**Table 1:** Proximate and Ultimate Analyses of Pine Bark on a Dry Basis^a^

Parameter	Standard Method	Value (wt % d.b.)
Volatile matter	ISO 18123:2015	74.6 ± 0.4
Ash content	ISO 18122:2015	1.2 ± 0.5
Fixed carbon	By difference	24.2 ± 0.4
C	ISO 16948:2015	53.2 ± 1.1
H	ISO 16948:2015	5.17 ± 0.7
N	ISO 16948:2015	0.50 ± 0.1
S	ISO 16994	0.03 ± 0.005
O	By difference	39.9 ± 1.4

a Values are reported as means ± 95% confidence intervals.

### 2.3 Operating Conditions and Procedures

A multiport outlet design allowed three distinct reactor configurations by selecting different ports for the vapor exit (Figure 2a). In the parallel flow (PF) configuration, vapors traveled in the same direction as the biomass and exited the reactor at the far downstream end (port 6). In the counterflow modes (CF), the vapor flow was reversed: in one case, denoted CF(2), vapors were forced to exit at an intermediate position (port 2); and in the other case, denoted CF(1), vapors exited at the feed inlet end (port 1). Each configuration thus created a different vapor–solid interaction environment, internal vapor residence time, and temperature profile. Prior to each run, the empty reactor was heated from ambient to the target temperature profile. Figure 2b shows the axial temperature set points of the five heater zones for the highest treatment temperatures (HTT) of 600, 700, and 800 °C. Throughout the heating, experimental, and cooling periods, the reactor was purged with nitrogen (3 L min^–1^ at STP; 0 °C and 1 atm) to maintain an inert atmosphere. O_2_ levels at the exhaust were monitored by an inline gas analyzer (SICK-Maihak S710). Once all zones stabilized within ±2 °C of their set points for at least 10 min and residual O_2_ became negligible (below 0.02 vol %), the biomass feeder was started. Each pyrolysis experiment processed ∼500 g of pine bark, fed via a screw feeder at ∼8.5 g min^–1^. A cold-flow test (feeding particles of the same size without heating) indicated a mean solid residence time of ∼19.6 ± 1.5 min in the reactor. The nitrogen gas flow was split between the feeder inlet (flow 11 in Figure 1) and the biochar outlet (flow 12 in Figure 1) to aid vapor transport and prevent it from entering the feeder and the char bin. In parallel-flow (PF) mode, 2 L min^–1^ was introduced with the feed and 1 L min^–1^ at the biochar discharge, whereas in counterflow (CF) modes, the flow directions were reversed (2 L min^–1^ at the biochar end and 1 L min^–1^ at the feeder) to drive the vapors upstream. These flow conditions were held constant for all tests. After all the solids entered the reactor (∼60 min), the feeder was stopped, and the reactor was purged with nitrogen for an additional ∼30 min (with heaters maintained). Temperature and pressure were recorded every 2 s, while gas composition was measured every 2 min throughout the experiments.

**Figure 2:**
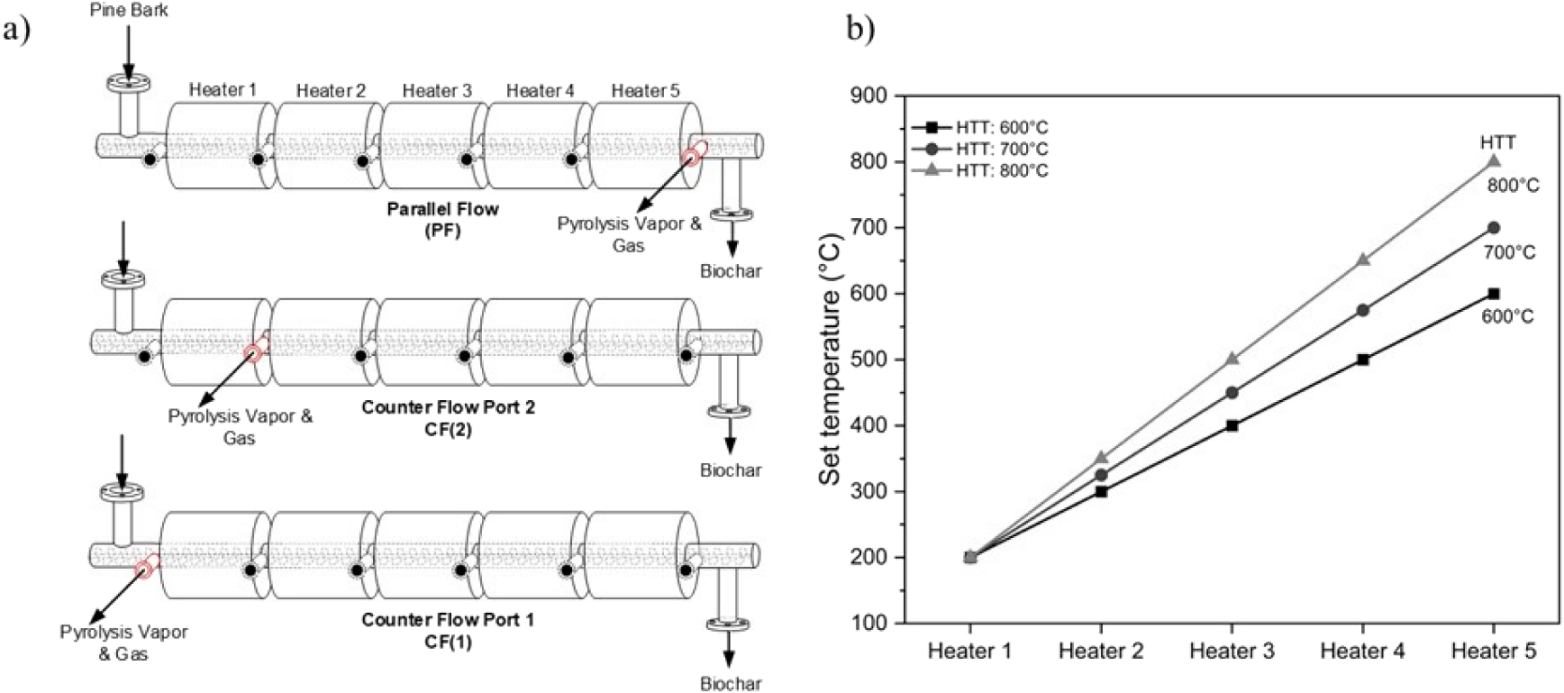
(a) Schematic figures of the three reactor outlet configurations used in this study and (b) axial temperature distribution within the auger reactor at different HTTs. Data represent the set values of heaters 1–5.

### 2.4 Product Collection and Analysis

#### 2.4.1 Biochar

Biochar leaving the reactor was collected in three stainless steel bins, separated by high-temperature ball valves. During the first 30 min of the experiments, while temperature, pressure, and gas composition were still stabilizing, all the valves to the char bins remained open, and all material produced during this period was collected in the bottom bin (see Figure 1—no. 5). At *t* ≈ 30 min, the bottom valve between the middle and the bottom bin was closed, and the biochar was collected in the middle bin (no. 4) between 30 and 70 min, representing biochar from fully developed, steady-state operation. Therefore, the biochar collected in the middle bin was used for all subsequent characterization. Ten minutes after the feeder was stopped (*t* ≈ 70 min), the top valve between the middle and top bins was closed, and any fines discharged from the reactor were retained in the top bin (no. 3). Once the reactor cooled down to room temperature, the three bins were removed from the reactor. Char collected in the bins was weighed and stored in bottles at room temperature. The total mass of char from the three bins was summed to give the total biochar mass. Following the cooldown, any residual char remaining inside the reactor tube was swept out using compressed air and included as residual char in the top bin. Proximate analysis was conducted according to the relevant standards, and elemental analysis (EuroVector EA3000) was performed. Both analyses were carried out on the biochar collected from the middle bin.

To quantify the relative increase in biochar yield between the parallel-flow and counterflow configurations, the total biochar yield (wt % of pine bark) was used. The relative change was calculated as:

1 $$\mathrm{Rel.}\;\Delta Y_{\mathrm{biochar}}=\frac{Y_{\mathrm{biochar}\left(\mathrm{CF}\right)}-Y_{\mathrm{biochar}\left(\mathrm{PF}\right)}}{Y_{\mathrm{biochar}\left(\mathrm{PF}\right)}}$$

Fixed carbon yield^5^ was employed to assess secondary char formation:

2 $$Y_{\mathrm{Fix-C}}=Y_{\mathrm{biochar}}\times \left(\frac{{\mathrm{Fix-C}}_{\mathrm{biochar}}}{100-Ash_{\mathrm{pine}\;\mathrm{bark}}}\right)$$

where Fix*C*_biochar_ is the fixed carbon content (wt % d.b.) of biochar and Ash_pine bark_ is the ash content (wt % d.b.) in pine bark. Furthermore, the energy conversion efficiency^5^ into biochar was evaluated using

3 $$\eta_{\mathrm{biochar}}=Y_{\mathrm{biochar}}\times \left(\frac{HHV_{\mathrm{biochar}}}{HHV_{\mathrm{pine}\;\mathrm{bark}}}\right)$$

where η_biochar_ is expressed in %, and HHV values are MJ kg^–1^.

#### 2.4.2 Multistage Condensation and Fractionation of Bio-oil

All condensable products were recovered by using a three-stage stainless-steel condensation system located downstream of the outlet port. The first two stages consisted of identical jacket-cooled condensers: the first was cooled with recirculating tap water at 10–15 °C, while the second was maintained at −10 °C using a glycol chiller. The third stage was a larger jacket-cooled (−10 °C, glycol) column filled with 25 mm × 25 mm 316L stainless steel Raschig rings, which provided an extended surface area for capturing the heaviest, low-volatility organic vapors. After each experiment, coolant circulation was maintained to ensure the complete drainage of condensates. Liquids were collected from sampling flasks beneath each condenser, weighed, and combined to determine the total bio-oil yield. The Raschig rings were also weighed to quantify the deposited oil, and this mass was added to the corresponding liquid fraction. It should be noted that the bio-oil collection efficiencies are expected to be lower than those of more advanced systems like spray condensers and electrostatic precipitators,^19^ especially due to the lack of collection mechanisms for highly volatile components such as benzene.

To further quantify and isolate the heavy, water-insoluble fraction of the collected bio-oil, a salt-assisted phase separation method (salting out) was applied.^20^ Following preliminary tests, MgSO_4_ was selected for its superior separation efficiency and reduced emulsification. A salt-to-oil mass ratio of 1:3 was used: 2.0 g of powdered MgSO_4_ (≥95%, VWR Chemicals) was added to 6.0 g (≈ 6 mL) of raw bio-oil in a glass tube, shaken vigorously by hand, and left to rest for 24 hours. This procedure produced two distinct phases: an upper water-insoluble, dark-colored, pyrolytic lignin-rich layer,^21^ referred to as heavy oil, and a lower aqueous phase. The mixture was filtered through a 0.45 μm filter to remove suspended solids, and the volume of the phases was measured by using a volumetric flask. Due to the high volatility of raw bio-oil, direct CHN analysis of the unfractionated sample was not feasible, as significant mass loss during sample handling introduced large uncertainties. However, analysis of the stable, viscous water-insoluble phase enabled reliable elemental characterization using the same elemental analyzer as for biochar.

#### 2.4.3 Gas Quantification

Permanent gases in the outlet stream were analyzed using an Agilent 490 Micro-GC system equipped with two different columns: CP-MolSieve 5A was used for H_2_, O_2_, N_2_, CH_4_, and CO, and PoraPlot U was used for CO_2_, C_2_H_4_, C_2_H_6_, and C_2_H_2_. Prior to each experiment, the system was calibrated using certified multigas calibration mixtures. Before analysis, the gas stream was conditioned using a PSS5 gas conditioning unit (M&C TechGroup), which included a gas cooler, a fine particle filter, a diaphragm pump, and a peristaltic pump for continuous removal of condensate. This ensured stable sampling conditions and avoided errors due to moisture or particle interference. Gas composition was analyzed every 2 min. The nitrogen content in the outlet gas was used as an internal tracer for flow normalization, under the assumption that all inlet N_2_ remains inert and that no additional N_2_ is generated. The instantaneous flow rate of each gaseous species *i*, *Q*_*i*_ *(t)*, at time *t* was calculated from the known total N_2_ flow rate, *Q*_N2_ *(t)*, and the GC-measured volumetric fractions of N_2_ and gaseous species *i*, *X*_N2_ *(t)* and *X*_*i*_ *(t)*, as:

4 $$Q_{i}\left(t\right){=Q}_{N2}\left(t\right)\times \frac{X_{i}\left(t\right)}{X_{N2}\left(t\right)}$$

To estimate the cumulative volume of each gas over the run, the time-resolved species flow data were numerically integrated using the composite trapezoidal rule as:

5 $$V_{i}=\overset{n-1}{\underset{k=0}{\sum }}\frac{Q_{i}\left(t_{k}\right)+Q_{i}\left(t_{k+1}\right)}{2}\times \left(t_{k+1}-t_{k}\right)$$

Here, *t*_*k*_ and *t*_*(k+1)*_ are successive sampling times, and *Q*_*i*_ *(tk)* is the instantaneous flow rate of species *i* at time *t*_*k*_. The result V*_i_* represents the total volume of species *i* produced during the run.

## 3 Results and Discussion

### 3.1 Reactor Performance

#### 3.1.1 Mass and Carbon Balances

The total product yield and material recovery varied significantly across the reactor configurations and temperatures. As shown in Figure 3, the parallel-flow (PF) setup (outlet at port 6) displayed noticeably higher unaccounted fractions with increasing trends along temperature rises, up to ∼20 wt % at HTT 800 °C. In comparison, the counterflow (CF) configurations (Ports 1 and 2) generally achieved mass closures within 90–95%, without a general trend against temperature changes. The relatively low mass balance closures of the PF configuration are either due to the ineffective condensation with the high temperature vapor inlet or due to the presence of high vapor pressure components, such as benzene (ca. 13 mbar at −10 °C), leading to a significant loss. However, the quantification of uncollected bio-oil fractions was considered to be outside the scope of this work.

**Figure 3:**
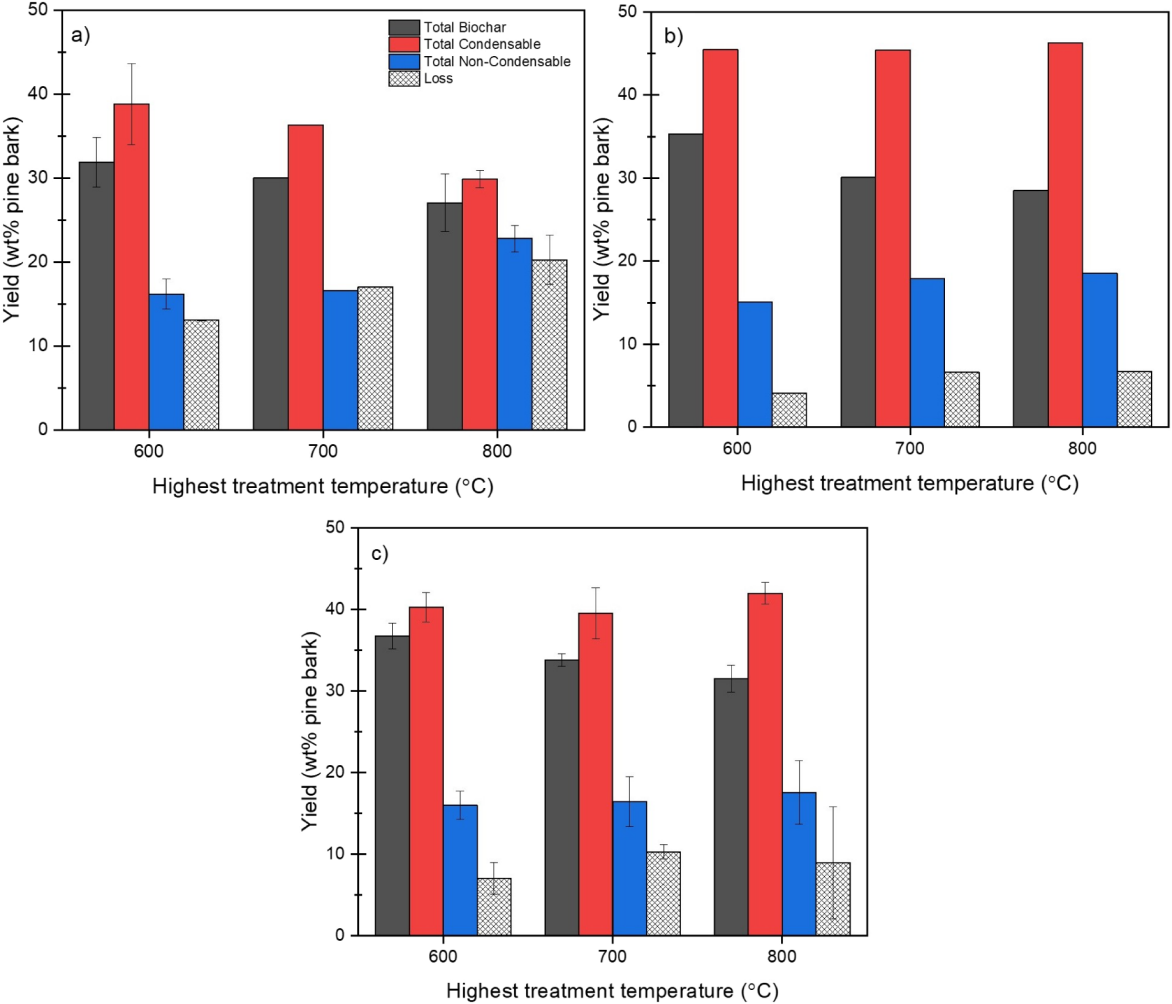
Product yields of total biochar, total condensable (bio-oil), and noncondensable gases under different reactor configurations: a) PF, b) CF(2), and CF(1) and HTTs, expressed as wt % of pine bark. Experimental losses calculated based on material balance. Error bars denote 80% confidence intervals for conditions where replicate runs were conducted (*n* = 2).

Figure 4 presents the total carbon balance, expressed as the percentage of feed carbon distributed among biochar, noncondensable gases, heavy bio-oil fraction (water-insoluble phase), and unaccounted fractions. Several key trends emerged from the carbon balance, revealing the interactions between reactor configuration and HTT. The PF configuration is consistently associated with the highest levels of unaccounted carbon, particularly at HTT 800 °C. Carbon retention in biochar increased from 48–59% in PF to 54–61% in CF(2) and reached the highest range in CF(1) (58–62%), indicating progressively more effective retention of carbon in the solid phase with enhanced vapor–solid interaction. Meanwhile, the highest carbon retention in heavy bio-oil was observed with the CF(2) configuration. Major sources of unaccounted carbon are expected to be in water-soluble bio-oil and light vapor bio-oil fractions that had not been collected in the condenser system. The latter would be especially important for the PF configuration, as the vapors likely were cracked into smaller molecules when passing through the highest temperature zone and exited from the reactor at the highest temperature.^9^

**Figure 4:**
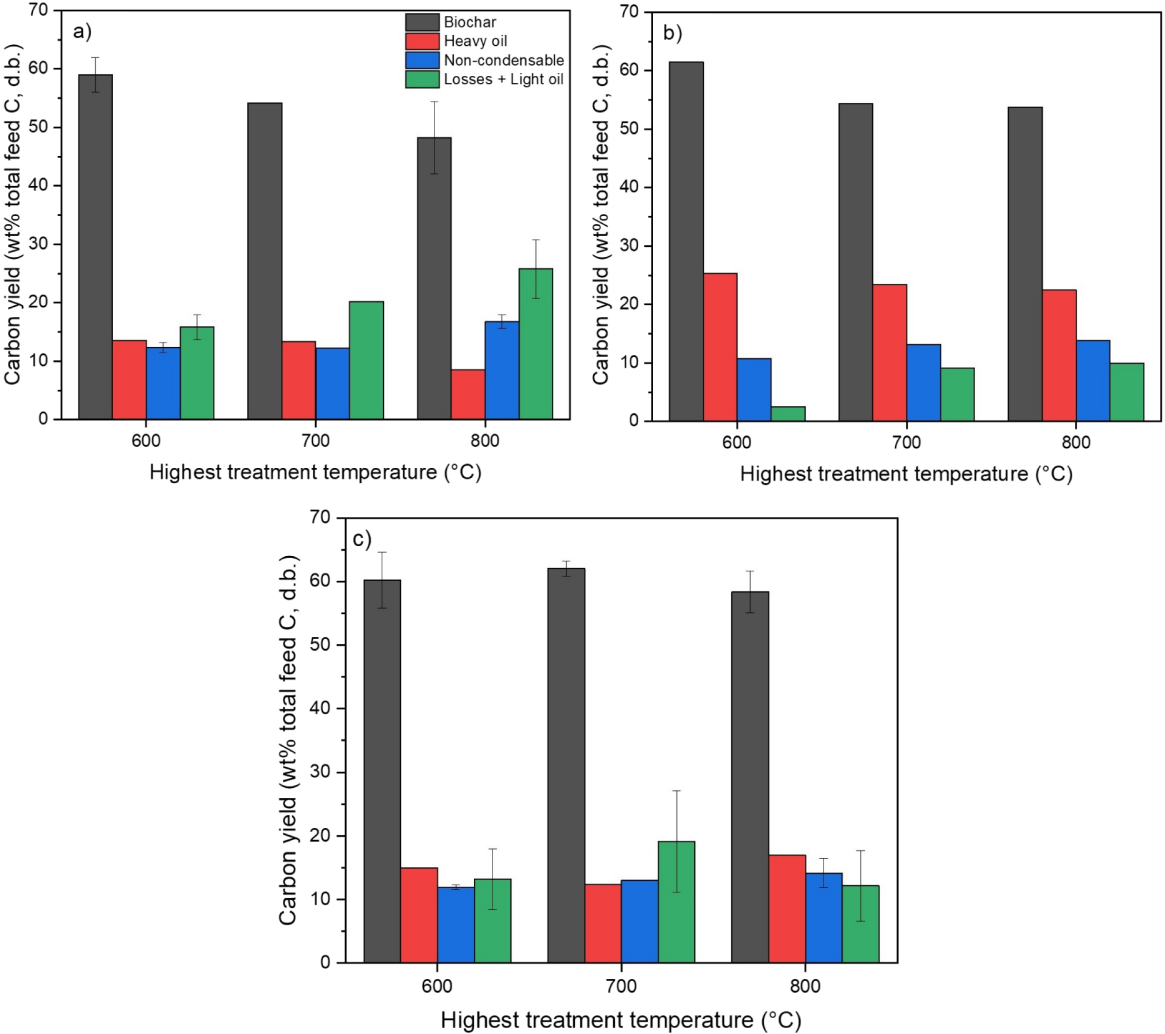
Carbon balance across different reactor configurations: a) PF, b) CF(2), and c) CF(1). Total carbon distribution is presented as a percentage of the total carbon content in dry pine bark, partitioned into biochar, noncondensable gases, heavy fraction of bio-oil, and unaccounted fractions (including losses).

#### 3.1.2 Temperature and Pressure Profiles

The axial temperature profile within the reactor was shaped by both the outlet port configuration and HTTs. Figure 5 presents the temperatures logged by six thermocouples (TC1–TC6) along the reactor body during the initial state before the feeding starts. Axial temperature profiles over time for each configuration and HTT are provided in the Supporting Information (Figure S1). These measurements reflect the internal gas-phase temperatures at key axial positions, both outside the heated zones (upstream of Heater 1, TC1, and downstream of Heater 5, TC6) and between heaters (TC2 to TC5). Due to the differences in nitrogen flow directions (2 L min^–1^ from right to left, 1 L min^–1^ from left to right for CF and the opposite for PF configurations), CF configurations showed slightly higher temperatures for TC2 to TC5 than PF configuration, while the temperature at TC6 was higher with PF configurations. Note that all of the temperature measurements between heaters (TC2 to TC5) show values below the set point of the heaters because the heating elements are located in the middle of the heaters, and heat loss from both ends of the heaters cannot be negligible, despite the insulation layers. Wall temperatures were additionally verified using a portable thermometer (Testo 915i) to ensure that the measured temperatures reached the actual set points. It was confirmed that the wall temperatures corresponded well with the target values.

**Figure 5:**
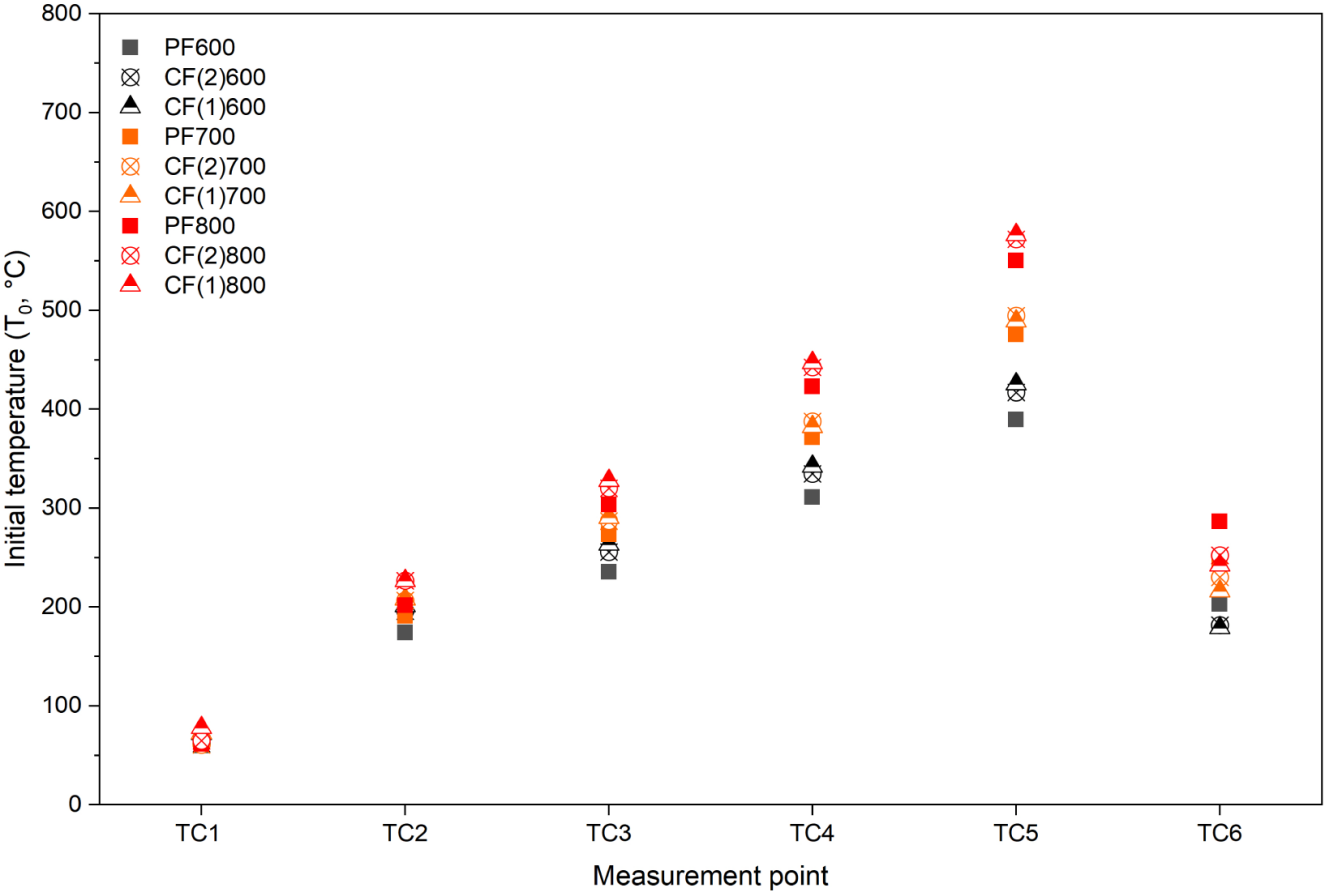
Axial temperature profile measured by six thermocouples (TC1–TC6) inside the reactor tube during the initial phase prior to biomass feeding. Data were recorded under nitrogen flow with an empty reactor bed.

Figure 6 further illustrates the local temperature change (Δ*T*) at each measurement point, calculated as the difference between the steady-state temperature (60 min after the feeding starts) and the initial temperature. Once feeding started and biomass, vapors, and biochar began flowing through the system, the temperature change diverged across configurations. TC1 and TC2 dropped across PF and CF(2) configurations due to cold incoming biomass. This effect was not observed in CF(1) since the heating effect from incoming vapors was also dominant. In all configurations, TC6 rose above the initial value due to exposure to hot vapors and biochar. For PF, vapor exit occurred at the hot end, leading to a more significant increase in TC6 (Δ*T* = 60.0–75.0 °C) than for CF. Meanwhile, TC2 dropped more considerably (Δ*T* = 45.0–65.0 °C) for PF as heating duties for cold incoming biomass were not compensated with counterflows of the hot gas stream like for CF. These shifts in PF produced the highest Δ*T* values among all configurations, especially at the reactor’s ends. In contrast, the CF configurations, particularly CF(1), maintained similar axial temperature profiles to initial conditions during steady-state operation. The temperature differences across zones remained within a moderate range, and cooling effects at TC1 were negligible due to heating effects by the counterflow of vapors. CF(2) exhibited similar temperature changes to CF(1) between TC3 and TC6 but became more similar to PF at TC1 and TC2. The gas flow for CF(2) conditions is parallel flow between TC1 and TC2 and counterflow between TC2 and TC6. The results indicate that the temperature distribution inside the reactor is not only affected by the wall temperature but also by heat carried by biomass/biochar and vapor flows.

**Figure 6:**
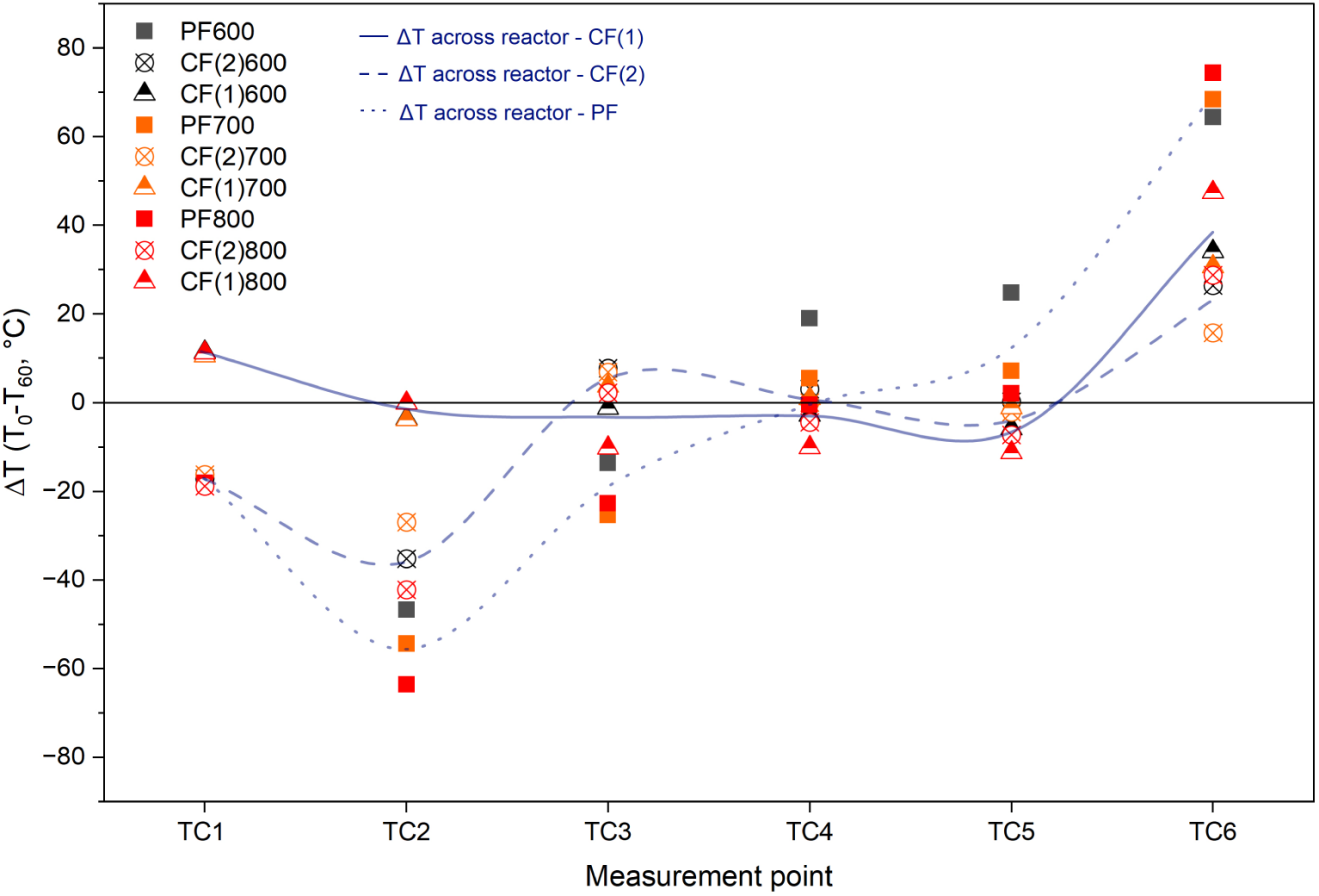
Local temperature changes (Δ*T*) at six axial positions (TC1–TC6) along the reactor during pyrolysis experiments. Δ*T* values were calculated as the difference between steady-state temperatures (measured at minute 60) and initial temperatures for each thermocouple.

Internal pressure measurements provide additional insight into the vapor flow behavior. The pressure profile measured along the reactor (see Supporting Information, Figure S2) reveals that internal pressure dynamics were strongly affected by the outlet port configuration. In the PF configuration, the pressure remained relatively uniform and low (≈0–20 mbar gauge) across all HTTs, indicating smooth vapor flow through the downstream outlet. Similarly, in the CF(1) configuration, the pressure remained stable (<10 mbar gauge), confirming negligible pressure buildup. In contrast, the CF(2) configuration showed a pronounced axial pressure gradient that intensified with increasing temperature: while P1 (above the outlet at port 2) remained low, P2 and P3 rose sharply, reaching up to ∼130 mbar at HTT 800 °C, indicating a potential resistance against free flow in downstream hot zones.

### 3.2 Effect of Reactor Configuration on Product Yield and Properties

#### 3.2.1 Biochar

The experimental findings clearly demonstrated that the reactor configuration significantly influences the yield of biochar (Figure 7). In general, CF configurations produced higher biochar yields compared to the PF case, with the most substantial enhancement observed in the CF(1) configuration, where the gas outlet is located upstream. This outcome is strongly linked to enhanced vapor–solid interactions and in situ vapor condensation on incoming biomass within the cold zone (Heater 1) of the reactor. In the CF(1) configuration, volatile vapors generated in hotter zones are forced to travel through the reactor progressively toward the cooler zones and come into physical contact with cold biomass before being removed from the system. This reverse contact not only facilitates the physical condensation of pyrolysis vapor onto the biomass surface but also promotes secondary char formation reactions when the condensates embedded on the biomass re-enter the hot zones. As shown in Figure 7, the relative biochar yield enhancement in the CF(1) setup is consistent and substantial at all HTTs, ranging between ∼12.5% and ∼15.5% in comparison with the PF configuration. In contrast, the CF(2) configuration showed a significant biochar yield increase only at HTT 600 °C (∼11.0%). This temperature-dependent behavior can be attributed to the localized temperature profile within the reactor. At HTT 600 °C, the temperature next to the vapor outlet remains relatively low (300 °C), enabling some vapors to condense onto the biomass matrix before exiting. However, at higher HTTs (700–800 °C), this temperature increases, reducing the fraction of condensable vapors and the residence time of vapors in the cold zones, thus reducing the likelihood of subsequent secondary char formation.

**Figure 7:**
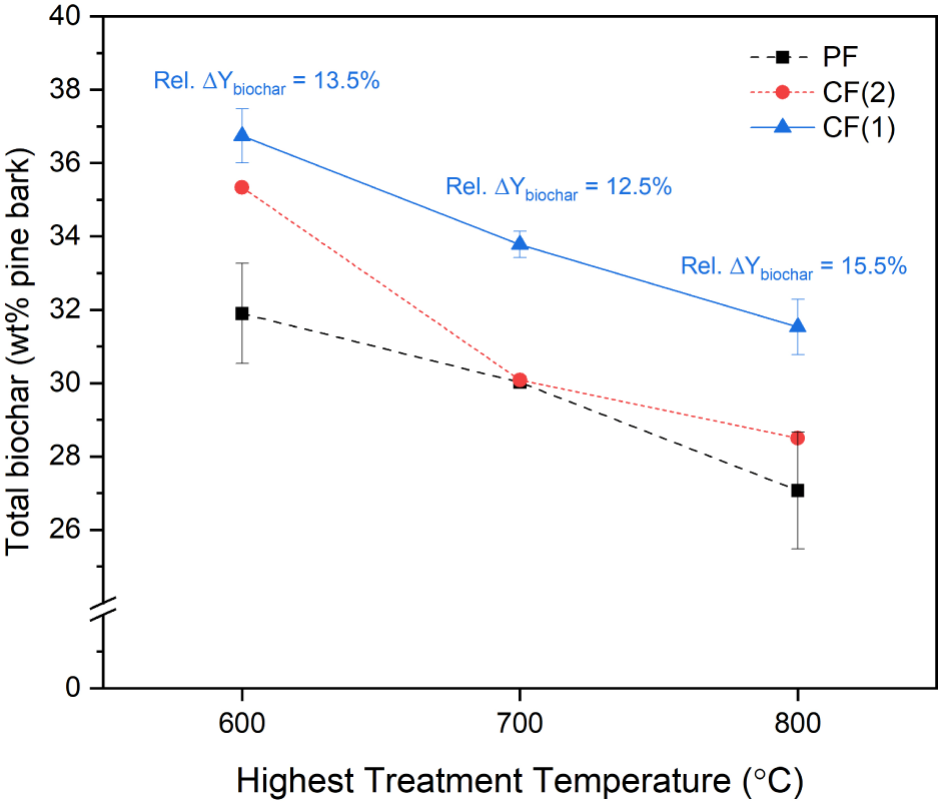
Total biochar yield (wt % of pine bark) at three HTTs under different reactor configurations and relative change in biochar yield (%) in CF+ compared to the PF baseline at each HTT.

Interestingly, while the biochar yield increases with CF configurations, the fuel composition (proximate analysis) of the biochar remains relatively consistent across different configurations at the same HTT (Table 2). Additionally, elemental analysis and the Van Krevelen diagram (Figure S3, Supporting Information) confirm that CF(1) biochar remains compositionally similar to PF at each HTT. This implies that the additional char formed in CF, particularly in CF(1), is compositionally similar to the PF biochar. However, one notable difference emerges in the ash content: CF(1) biochar consistently exhibits lower ash content than those from CF(2) and PF at the same HTT. This observation supports the hypothesis that a fraction of the biochar is formed via in situ condensation and subsequent polymerization of vapors. This so-called secondary char is organically enriched and thus dilutes the ash concentration of the total solid.

**Table 2:** Proximate Analysis Results and Higher Heating Values (HHVs) of Biochars Produced at Different HTTs and Reactor Configurations^a^

		Composition of Biochar (wt % d.b.)							
Reactor Configuration	HTT, °C	VM	Ash	Fixed-C	C	H	N	O	HHV, MJ/kg
PF	600	17.3 ± 0.06	5.0 ± 0.27	77.3 ± 0.27	85.3 ± 1.56	2.9 ± 0.62	0.3 ± 0.34	6.5 ± 1.73	33.14
	700	8.3 ± 0.20	4.8 ± 0.01	86.6 ± 0.20	89.7 ± 0.37	2.2 ± 0.16	0.45 ± 0.23	2.8 ± 0.46	32.97
	800	5.4 ± 0.08	5.4 ±0.02	89.0 ± 0.08	91.9 ± 0.82	1.6 ± 0.10	0.20 ± 0.18	1.0 ± 0.84	33.14
CF(2)	600	18.0 ± 0.65	3.8 ± 0.12	78.2 ± 0.66	85.50 ± 2.0	3.10 ± 0.26	0.20 ± 0.08	7.50 ± 2.02	31.97
	700	10.0 ± 0.92	4.6 ± 0.11	85.10 ± 0.93	89.10 ± 0.27	2.30 ± 0.16	0.45 ± 0.29	3.60 ± 0.59	32.76
	800	5.7 ± 0.61	5.4 ± 0.17	88.8 ± 0.64	92.70 ± 1.66	1.60 ± 0.03	0.10 ± 0.02	0.20 ± 1.67	33.62
CF(1)	600	17.5 ± 0.12	3.9 ± 0.24	78.30 ± 0.27	85.90 ± 1.91	3.20 ± 0.27	0.50 ± 0.29	6.53 ± 1.96	32.41
	700	10.2 ± 0.62	4.2 ± 0.07	85.20 ± 1.82	89.90 ± 0.74	2.40 ± 0.23	0.40 ± 0.32	3.10 ± 0.94	32.86
	800	5.2 ± 0.10	4.7 ± 0.07	89.0 ± 0.12	93.1 ± 1.51	1.50 ± 0.14	0.5 ± 0.36	0.20 ± 1.56	32.41

a Data are reported as mean ± standard deviation (dry basis).

This observation is further supported by the relationship between the fixed-carbon yield and energy conversion efficiency presented in Figure 8. A distinct improvement in both parameters is observed for the CF(1) configuration compared to PF across all HTTs. CF(1)­600 showed the highest energy conversion efficiency into biochar (≈65.0%) and fixed-carbon yield (30.0%). CF(2) exhibits enhanced biochar characteristics only at the lowest HTT, supporting the previous findings related to its high relative biochar yield at the same HTT. Lastly, PF800 sample shows a fixed-carbon yield nearly equal to that of the feedstock, though with a relatively large standard deviation. Almost all configurations exhibit fixed-carbon yields above unity relative to the feed, indicating secondary char formation under both parallel and counterflow conditions. Nevertheless, the enhancement observed in CF(1) highlights its superior secondary reaction efficiency.

**Figure 8:**
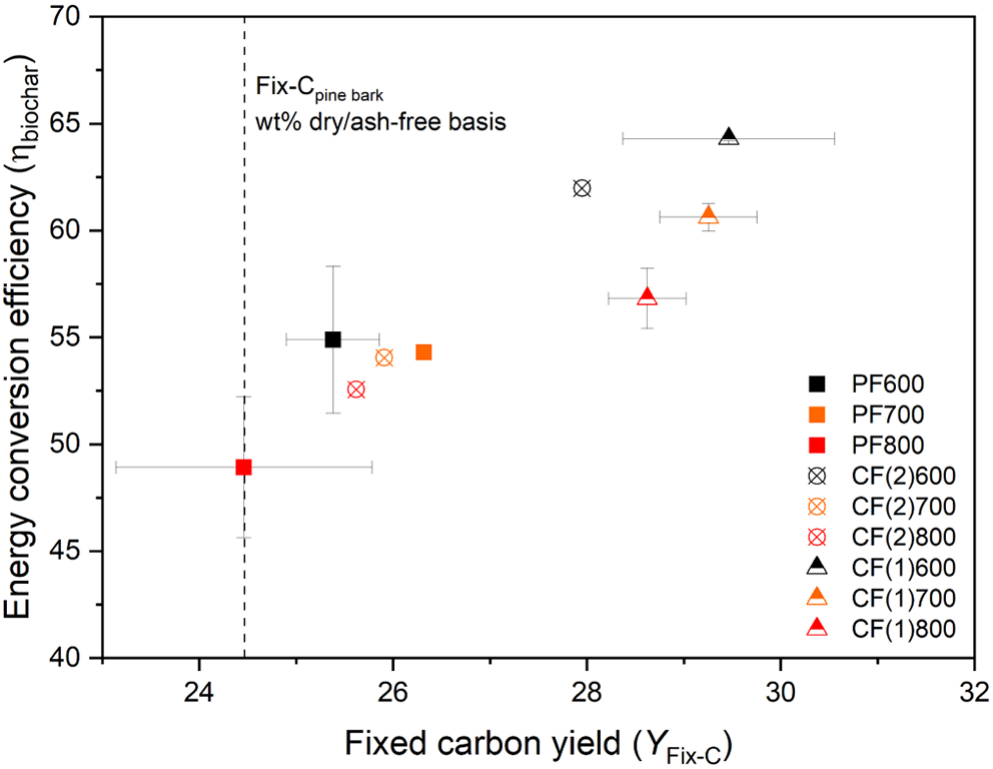
Relationship between the fixed carbon yield (*Y*_Fix‑C_) and energy conversion efficiency (η_biochar_) for different reactor configurations and HTTs.

#### 3.2.2 Bio-oil

The bio-oil yield and composition were strongly governed by the vapor outlet port configuration. As previously shown in Figure 3, among all configurations, the highest bio-oil yields were obtained in the CF configurations. In CF(2), where vapors recirculated through intermediate hot zones and exited before reaching the cold zone, bio-oil recovery was maximized across all HTTs (45.0–46.0 wt %). This configuration allowed internal cooling without reaching a temperature too low, which might otherwise trigger in situ condensation. CF(1), where vapors circulate and pass through the coldest zone prior to exit, also yielded higher bio-oil recovery (40.0–42.0 wt %) than PF (30.0–37.0 wt %), though slightly lower than CF(2). The decline in CF(1) yield is attributed to vapor–solid interactions in the cold zone, where heavy volatiles likely condensed or deposited onto biomass particles, promoting secondary char formation (see the previous section). The lowest bio-oil yields were consistently observed in the PF, particularly at higher HTTs. In this mode, vapors exited directly from the hot zone. This resulted in inefficient recovery of condensable vapors for the current condenser system, as confirmed by visible tar deposition in the lines downstream of the condenser system.

The distribution of bio-oil collected between the condensers also varied with the configuration (Figure 9). In all tests, most of the oil was recovered from condenser 1, followed by condenser 2, and finally condenser 3. The relative contribution of condenser 3 was highest in CF(2), indicating that the highest amount of nonvolatile heavy oil left the reactor as vapor. In the PF configuration, the low-level recovery of bio-oil coincided with higher outlet vapor temperatures, suggesting that heavy oil fractions were either thermally cracked or deposited postcondensation. The relatively low and consistent contribution of condenser 3 in CF(1) suggests that the heavy oil vapor stream was efficiently condensed on the incoming biomass surface and converted to secondary char rather than being condensed externally. The results indicate the potential to shift the selectivity between char and bio-oil by extracting the pyrolysis vapor at different temperature ranges under counterflow conditions.

**Figure 9:**
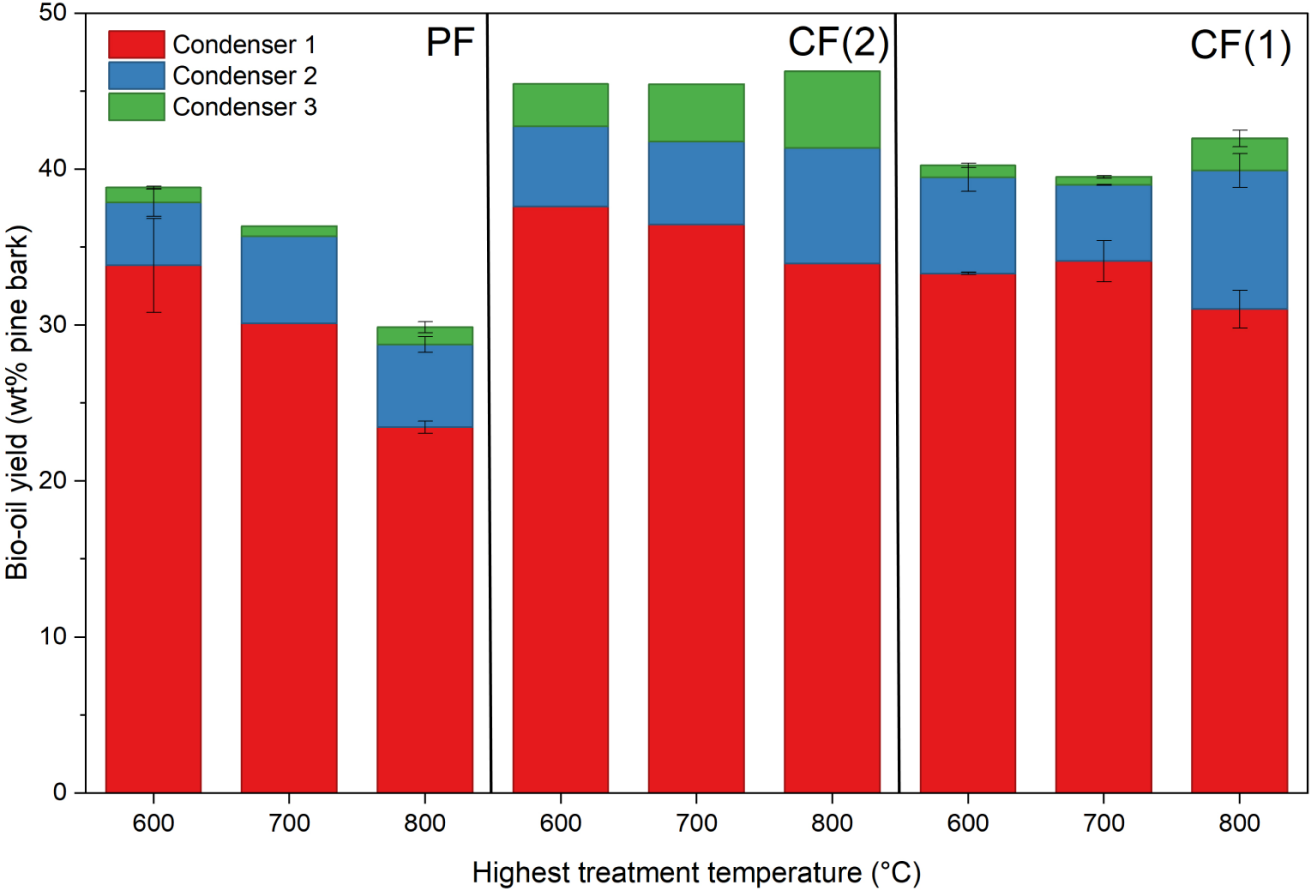
Bio-oil yield distribution across the three-stage condensation system for all reactor configurations and HTTs. Error bars denote standard deviation for conditions where replicate runs were conducted (*n* = 2).

Heavy oil (water-insoluble PL-rich phase) fractions and their elemental compositions provide additional insights. The volumetric share of water-insoluble fractions in the oils collected from condensers 1 and 2 remained relatively stable within each configuration group: ∼25–30 vol % for PF, ∼35–45 vol % for CF(2), and ∼25–35 vol % for CF(1). The highest water-insoluble recovery was observed in CF(2). Elemental analysis of these fractions showed modest variations in carbon and hydrogen content across conditions (48.0–49.0  wt % C, ∼6.0 wt % H).

#### 3.2.3 Noncondensable Gas

The production of noncondensable gases (NCG), CO, CO_2_, H_2_, CH_4_, and C_2_–C_3_ hydrocarbons, is a clear severity indicator of char carbonization and vapor secondary cracking. As seen earlier (Figure 3), NCG yields increased with temperature in all cases, but the reactor configuration also affected the composition of the gas. Figure 10 presents the NCG yields for major species (H_2_ and CH_4_) for each configuration at various HTTs (CO and CO_2_ yields are provided in Figure S4). The focus was on H_2_ and CH_4_ as their yields are strongly tied to secondary reactions, tar cracking, and carbonization of char.^22^ In PF, H_2_ yields were relatively low: ∼0.005 N m^3^/kg pine bark at HTT 600 °C, rising to ∼0.018 N m^3^/kg at HTT 800 °C. The limited H_2_ indicates that primary tar cracking, char carbonization, and water gas and water–gas shift reactions were modest, consistent with vapors not staying long in hot zones. In contrast, the CF modes produced significantly more hydrogen. CF(1) H_2_ yield reached ∼0.037 N m^3^ kg^–1^ at HTT 800 °C, roughly double that of PF. This can be attributed to prolonged exposure of heavy volatiles to the high-temperature zones in the reactor and the extended residence time through repeated internal recirculation through devolatilization and condensation. This favors tar reforming toward carbonization into char that generates H_2_. Similarly, CH_4_ showed a pronounced increase with CF. CH_4_ yields were the lowest (∼0.009–0.012 N m^3^/kg across HTT 600–800 °C) under PF, whereas CF(1) yielded up to ∼ 0.02–0.025 N m^3^/kg at HTT 800 °C. Methane in pyrolysis gas primarily comes from methoxy and methyl group fragmentation (e.g., demethoxylation of guaiacols and breakage of side chains in lignin units). The higher CH_4_ in the CF configurations suggests more extensive breakdown of lignin-derived vapors, which aligns with the increased conversion of those vapors to either biochar, light oil, or gas. Indeed, the same conditions that produce secondary char also tend to release small molecules, such as CH_4_ and H_2_. These trends are in line with general biomass pyrolysis behavior reported in literature: more severe vapor-phase processing (longer residence time in hot conditions) yields more H_2_/CH_4_ at the expense of tar.^23^

**Figure 10:**
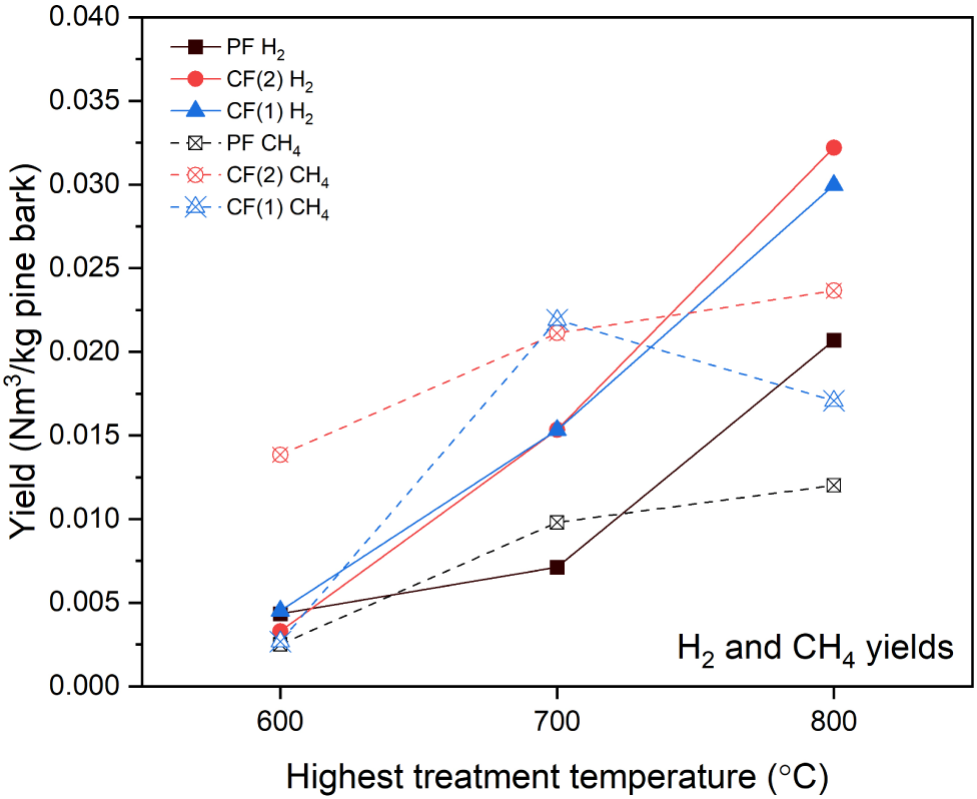
Yield of H_2_ and CH_4_ produced across different reactor configurations and HTTs.

## 4 Conclusions

This study demonstrated that in situ recirculation of pyrolysis vapors, governed by the reactor outlet configuration, significantly influences product yields and carbon retention in biochar during the pyrolysis of pine bark in an auger reactor. A comparison of parallel-flow (PF) and counterflow (CF) configurations at varying highest treatment temperatures (HTTs) showed that the internal flow directions of pyrolysis vapors and temperature ranges play a decisive role in regulating in situ vapor–solid interactions, leading to different condensation and secondary pyrolysis reaction pathways.

The CF configurations, where vapors traverse the biomass bed in reverse through the coldest zone, promoted intensive vapor–solid interaction, resulting in efficient heavy oil condensation inside the reactor. This enhanced secondary char formation and increased relative char yield, fixed carbon yield, and energy conversion efficiency into biochar. In contrast, PF operation resulted in the lowest char yield and inefficient condensation by a conventional condenser system, leading to the highest unaccounted carbon losses in this study.

The comparison of the two CF configurations implies that vapor–solid interaction at a sufficiently low temperature (below 200 °C in this study) is required to efficiently capture the heavy-oil fraction on incoming biomass and fully utilize the benefit of in situ heavy-oil recirculation. Further parametric studies on the temperature distribution in the vapor–solid contact zones may clarify the conditions necessary to efficiently capture heavy oil selectively. In addition, the detailed characterization of collected bio-oil, such as the molecular size distribution and functional groups, would enhance our understanding of secondary vapor/oil cracking occurring inside the auger reactor.

Overall, the results highlighted that strategic control of vapor flow configurations within the reactor can serve as an effective tool to tailor product distribution toward selectively enhancing solid carbon recovery. These insights offer a valuable design basis for the development of scalable pyrolysis systems aimed at optimizing the biochar yield and quality while minimizing the formation of problematic heavy bio-oil fractions. However, it should be noted that the effects of vapor flow configurations may not be as significant in other reactor types such as rotary kilns as in auger reactors, because the presence of freeboard above solid beds results in a lesser extent of vapor–solid contact.

## Supplementary Material



## Notes

Additional figures showing axial temperature and pressure profiles (Figures S1 and S2), Van Krevelen diagram of biochar (Figure S3), and CO/CO_2_ yields across configurations and HTTs (Figure S4) (PDF)
