# Supplementary material for: Selective Product Enhancement in an Auger Reactor: Pyrolysis of Pine Bark through In Situ Recirculation of Pyrolysis Vapors
Source: Energy Fuels. 2026 Feb 19;40(9):4693–703. doi: 10.1021/acs.energyfuels.5c06037 (PMC12969260; doi:10.1021/acs.energyfuels.5c06037)
Supplement: Supplementary file 1 [file ef5c06037_si_001.pdf]

Supporting Information

Selective Product Enhancement in an Auger Reactor: Pyrolysis of Pine Bark through In Situ Recirculation of Pyrolysis Vapors

Yusuf Tolunay Kilic [†,*], *Marcelo Dal Belo Takehara* [†], *Øyvind Skreiberg* [‡], *Kentaro Umeki* [†]

[†] Division of Energy Science, Luleå University of Technology, Luleå SE-97187, Sweden

[‡] Department of Thermal Energy, SINTEF Energy Research, Trondheim NO-7465, Norway

This document contains 5 pages and 4 figures.

Change in axial temperature profile. The change on axial temperature profile during pyrolysis is presented in Figure S1 for each reactor configuration PF, CF(1), and CF(2) and highest treatment temperature (HTT). The plots capture the thermal gradients along the reactor axis (TC1 to TC6) and highlight the influence of vapor flow direction.

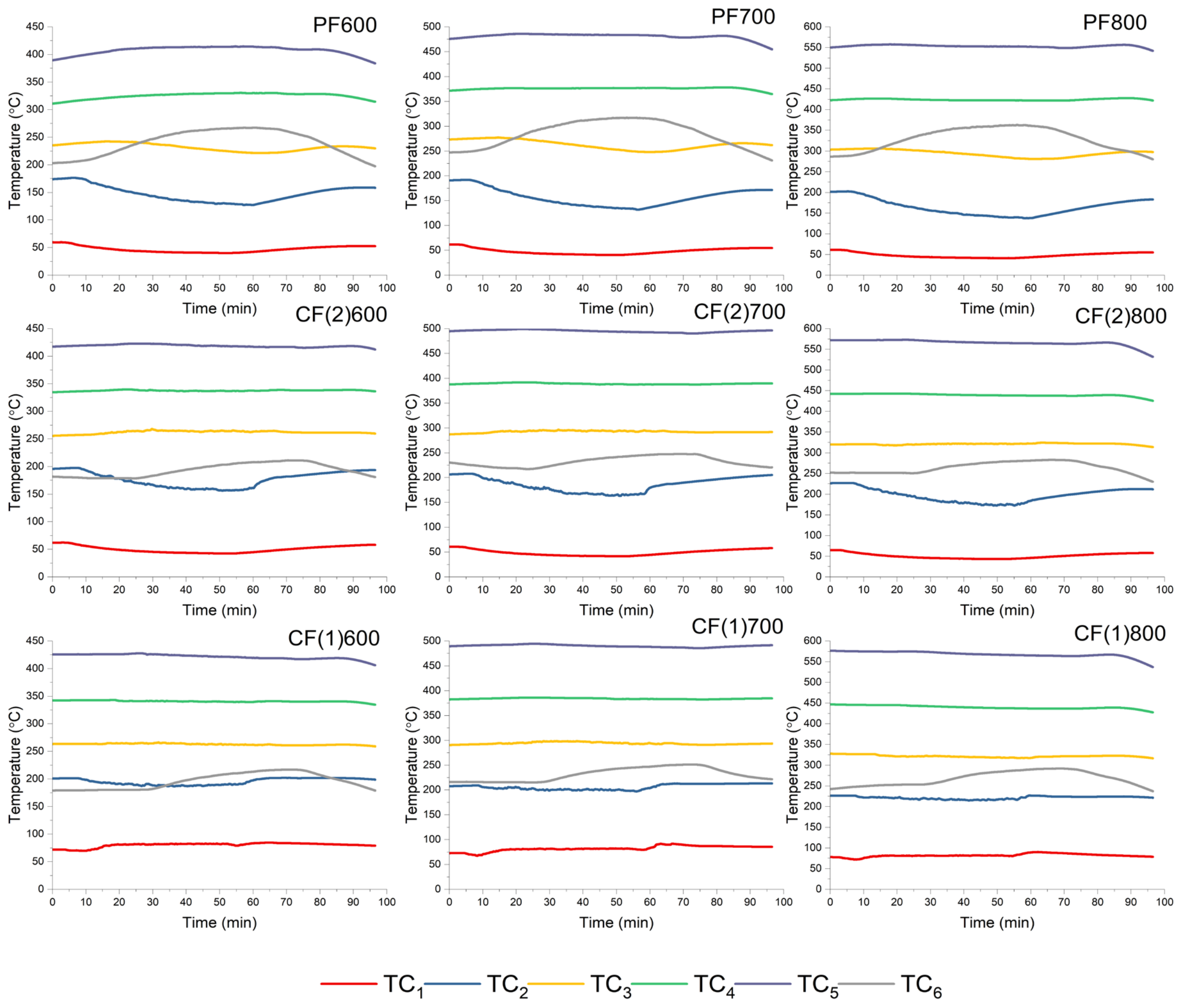

Figure S1. Axial temperature profile measured by six thermocouples (TC1–TC6) inside the reactor tube during pyrolysis (from initial condition to end of the experiment).

Change in axial pressure profile. Pressure profile during the steady-state pyrolysis period (20–60 min) for all configurations and HTTs is shown in Figure S2. PF and CF(1) configurations exhibited relatively low and uniform pressure distributions, indicating smooth flow regime along the reactor. In contrast, CF(2) consistently showed pronounced axial pressure gradients, with substantial buildup at P2 and P3. Notably, elevated pressure readings at these positions persisted during the post-pyrolysis (cooling phase), suggesting possible in-situ condensation of condensables or tars near the pressure transducer connection points in the downstream of the reactor.

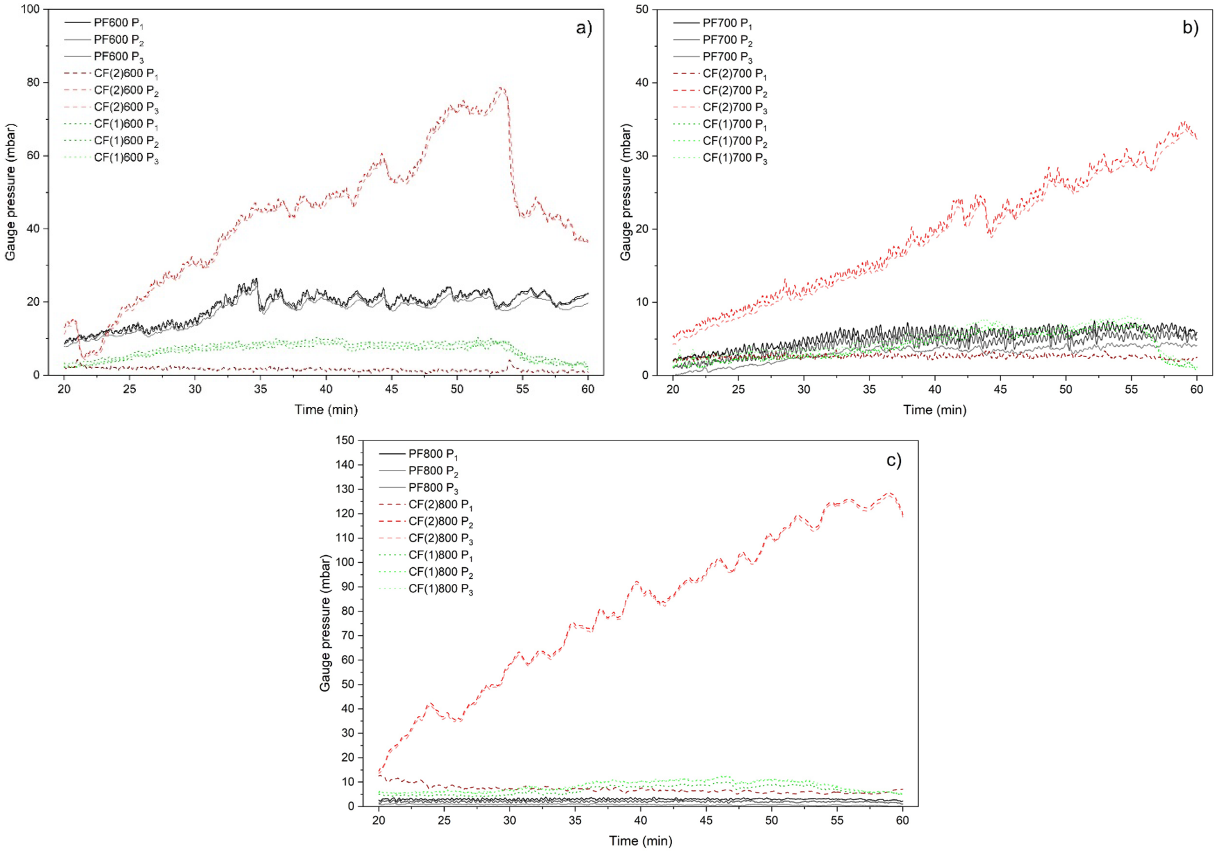

Figure S2. Gauge pressure profiles recorded at three axial positions (P1, P2, P3) during pyrolysis at HTTs of (a) 600, (b) 700, and (c) 800 °C under varying reactor configurations: PF, CF(2), and CF(1).

Van Krevelen diagram The configuration-dependent formation of secondary char is emphasized in the van Krevelen diagram (Figure S3), where the H/C and O/C ratios of biochar cluster by HTT rather than by outlet port. This reinforces that while configuration modulates yield, the thermal severity (HTT) remains the dominant factor shaping elemental composition.

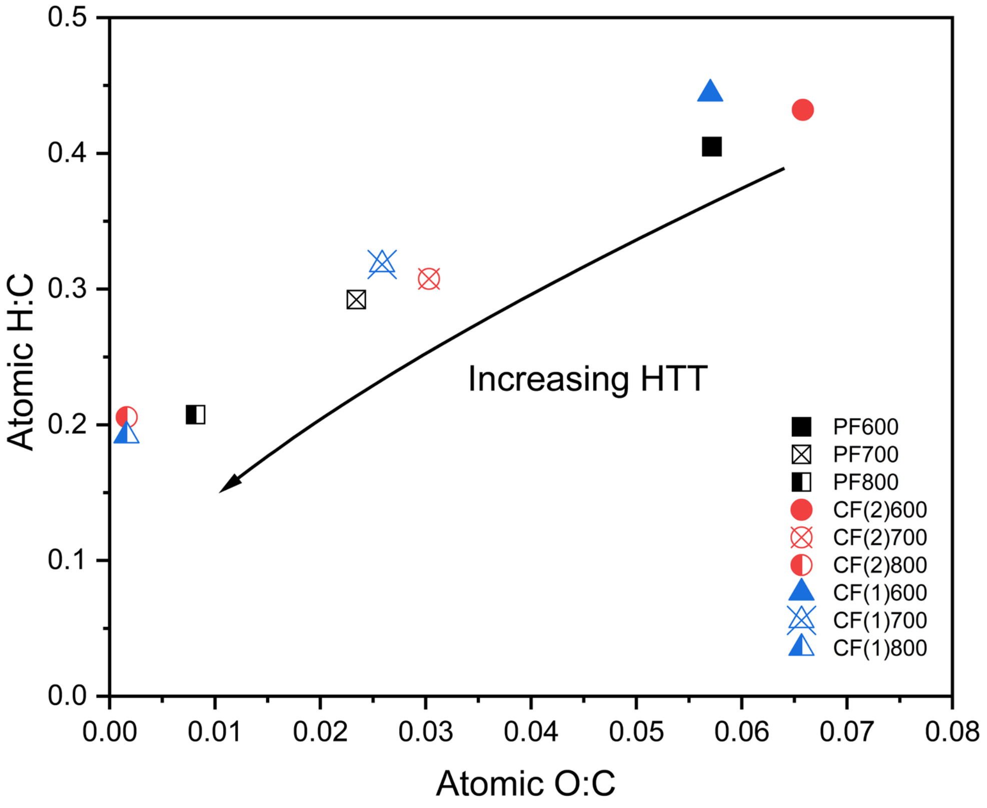

Figure S3: Van Krevelen diagram showing the atomic H:C and O:C of biochar samples across all reactor configurations and HTTs. (Atomic mass of H: 1.008, C:12.011, O: 15.999)

CO and CO_2 yields. Figure S4 shows the CO and CO_2 yields from all configurations at different HTTs. The CO_2 yield was relatively higher than other species all configurations. In the PF configuration, CO yields gradually decrease with increasing HTT. For CF(1) and CF(2), CO yields increased with HTT, particularly in CF(2), where the highest CO yield is observed at 800 °C.

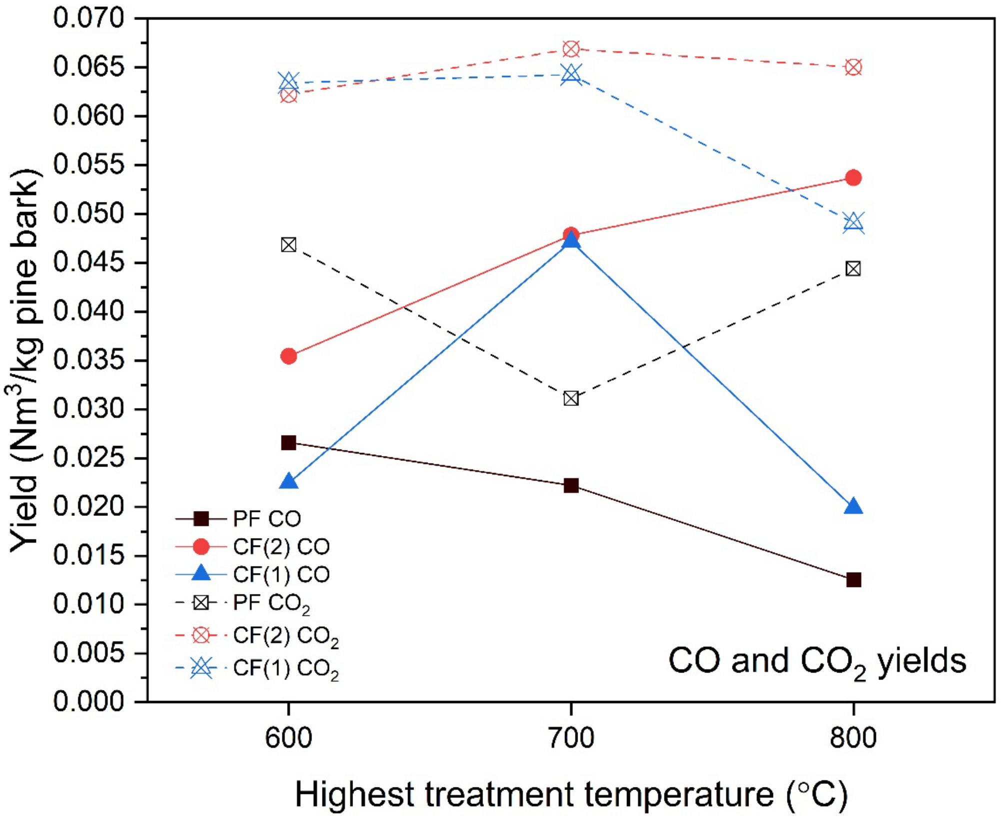

Figure S4: The yields of CO and CO_2 produced across different reactor configurations and HTTs.
